# A Proline-Rich Domain in the Genotype 4 Hepatitis E Virus ORF3 C-Terminus Is Crucial for Downstream V^105^DLP^108^ Immunoactivity

**DOI:** 10.1371/journal.pone.0133282

**Published:** 2015-07-15

**Authors:** Heng Wang, Fangxiao Ji, Huanbin Liang, Honglang Gu, Zhangyong Ning, Rongchang Liu, Guihong Zhang

**Affiliations:** 1 College of Veterinary Medicine, South China Agricultural University, Guangzhou, Guangdong Province, 510642, China; 2 Key Laboratory of Comprehensive Prevention and Control for Severe Clinical Animal Diseases of Guangdong Province, Guangzhou, Guangdong Province, 510642, China; 3 MOA Key Laboratory of Animal Vaccine Development, Guangzhou, Guangdong Province, 510642, China; National Institute for Viral Disease Control and Prevention, CDC, China, CHINA

## Abstract

The hepatitis E virus (HEV) is responsible for serious viral hepatitis worldwide. Animals are considered a reservoir of HEV, particularly pigs. While HEV infection in pigs and dogs is always asymptomatic, the virus causes high death rates in patients with pre-existing chronic liver disease and pregnant women in developing countries. HEV open reading frame 2 (ORF2) has been used as a diagnostic target to detect specific antibodies against HEV in serum samples. Recent research has additionally supported the potential utility of the ORF3 protein as a target in serum anti-HEV detection. However, the epitope distribution of ORF3 protein remains ambiguous. In the current study, we showed that continuous amino acid motif, VDLP, at the C-terminus of genotype 4 HEV ORF3 is a core sequence of the ORF3 protein epitope. Moreover, cooperative interaction with upstream elements is essential for its immunoactivity. Three proline residues (P99, P102 and P103) in the upstream proline-rich domain exerted significant effects on the immunocompetence of VDLP. ELISA results revealed that SAPPLPPVVDLP and SAPPLPPVVDLPQLGL peptides containing the identified VDLP epitope display weaker reactions with anti-HEV serum than the commercial ELISA kit. Our collective findings provide valuable information on the epitope distribution characteristics of HEV ORF3 and improve our understanding of the influence of the proline-rich domain on the immunoactivity of downstream amino acids in the C-terminal region.

## Introduction

HEV is an important pathogen responsible for serious epidemic hepatitis E worldwide, especially in developing countries with poor public health and sanitation infrastructures [1]. Human HEV is divided into four genotypes. Genotypes 1 and 2 are believed to specifically infect humans whereas genotypes 3 and 4 infect humans and other animals [2,3,4,5]. Genotype 4 is mainly prevalent in Asia [6,7]. In China, serum positive for HEV antibodies has been identified in humans and swine herds [8,9,10], as well as cows, goats, horses and pet dogs [11]. The primary route of transmission is suspected as fecal-oral, usually through HEV-contaminated water or food (raw and undercooked meat or liver) [12,13,14]. While HEV infection in pigs and dogs is always asymptomatic, high death rates have been reported in patients with pre-existing chronic liver disease and pregnant women in developing countries [15].

HEV belongs to the genus Hepevirus of the family Hepeviridae. The virus has a single-stranded, positive-sense RNA genome ~7.2 kb in length, and includes three partially overlapping open reading frames (ORFs). ORF1 encodes the nonstructural protein [16]. Recent studies have shown that papain-like cysteine protease (PCP) and X domain (macrodomains) encoded by ORF1 are putative interferon antagonists [17]. ORF2 encodes the viral capsid protein containing the neutralization epitopes. The minimal neutralization epitopes have been identified within residues 458–607 in the protruding region of the capsid protein [18,19,20,21]. Thus, ORF2 is considered to have significant potential for application in vaccine development [22,23,24] and diagnosis. ORF3 encodes a 113–114 residue multifunctional protein required for activating extracellularly regulated kinase [25], releasing virus, facilitating infection [26] and increasing expression of glycolytic enzymes [27]. Earlier investigations strongly suggest that a number of ORF3 peptides have good antigenicity and are highly sensitive [28], supporting the utility of ORF3 as a potential candidate for detecting specific antibodies against HEV in serum samples.

The main objective of this study was to elucidate the characteristics of epitopes of the HEV ORF3 protein. The continuous amino acid motif, VDLP, in the C-terminal region was identified as a core site of the epitope using the Phage Display Peptide Library. Notably, three prolines at positions 99, 102 and 103 of the upstream proline-rich domain exerted a significant effect on the immunocompetence of VDLP. Our findings provide valuable information on the epitope distribution characteristics of HEV ORF3 and improve our understanding of the influence of the proline-rich domain on immunoactivity of the downstream sequence in the C-terminal region of ORF3.

## Materials and Methods

### Expression and purification of His-tagged HEV ORF3 protein

The recombinant plasmid, pcDNA3.1-ORF3, containing the full-length ORF3 gene of genotype 4 HEV derived from pig (Accession No. JX855794, constructed and stored in our laboratory), was used as a template to amplify a truncated ORF3 gene fragment using the sense primer P1: 5ʹ-CCCAAGCTTATGGAGATGCCACCATGCG-3ʹ and antisense primer P2: 5ʹ-CCGGATATCTACGGCGAAGCCCCAGC-3ʹ containing *Hind*III and *EcoR*V restriction sites, respectively. The PCR conditions were as follows: 95°C for 3 min, 30 cycles of 95°C for 30 s, 58°C for 30 s, 72°C for 45 s, and final extension at 72°C for 5 min. Purified PCR products were digested with *Hind*III and *EcoR*V and inserted into the corresponding restriction sites of pET-32a plasmids. The generated constructs were transformed into *Escherichia coli* (*E*.*coli)* strain BL21 (DE3) pLysS, and ORF3 expression induced with isopropyl-β-d-thiogalactoside (IPTG) at a final concentration of 1 mM at 37°C. Bacterially expressed protein was identified using sodium dodecyl sulfate-polyacrylamide gel electrophoresis (SDS-PAGE).

Bacterial cell pellets were harvested from 20 mL culture medium after induction at 37°C, suspended in 4 mL phosphate-buffered saline (PBS), and sonicated on ice. Total cellular proteins were partitioned into soluble and insoluble fractions via centrifugation at 12,000 r/min at 4°C for 15 min and assessed via SDS-PAGE.

His-tagged ORF3 proteins were purified using Ni sepharose 6 Fast Flow affinity medium, according to the manufacturer’s instructions (GE Healthcare AB, Stockholm, Sweden). Purified proteins were analyzed using SDS-PAGE and western blot.

### Preparation of specific monoclonal antibodies

Six 5-week-old female Balb/c mice were subcutaneously immunized with 0.5 mg His-tagged ORF3 protein emulsified with an equivalent amount of complete Freund's adjuvant (Sigma-Aldrich, USA). Mice received two booster immunizations with the ORF3 protein plus Freund's incomplete adjuvant at 14-day intervals. At 7 days after the third immunization, blood samples were drawn to detect antibody titers using indirect ELISA. Mice with the highest serum titers, which were sacrificed by CO_2_ inhalation, were selected for subsequent fusion. Three days before fusion, selected mice were administered an additional intraperitoneal booster with the same antigen diluted in PBS.

Fusion of SP2/0 myeloma cells with spleen cells isolated from selected mice was performed using standard methodology [29]. Five days later, supernatant fractions incubated in 96-well plates were screened using indirect ELISA. Hybridoma cells in positive wells were cloned via limiting dilution screening in aminopterin-free selection medium, in which wells were coated with purified His-tagged ORF3 and 6 x His tag proteins, respectively. Next, hybridoma cells were injected into BALB/c mice to produce ascites. At 7 days post-injection, ascites were extracted and centrifuged at 1500 x *g* for 20 min at 4°C. Supernatant fractions were purified using liquid affinity chromatography.

Animal experiments were conducted in keeping with the recommendations in the Guide for the Care and Use of Laboratory Animals of the Ministry of Science and Technology of the People’s Republic of China. The present animal study was approved by the Animal Experimental Ethics Committee of the South China Agricultural University (Approval number 2014–07).

### Detection of HEV ORF3 expression in baby hamster kidney (BHK) cells using prepared monoclonal antibodies

BHK cells were seeded in 12-well plates (1 x 10^5^ cells/well) the day before transfection. Prior to transfection, cells were washed twice with serum-free OptiMEM (Gibco Life Technologies, Grand Island, NY, USA). Next, the cells were transfected with pcDNA3.1-ORF3 plasmids using Lipofectamine 2000 (Invitrogen) according to the protocol instructions.

After transfection for 48 h, cells were fixed in 4% (w/v) formalin in PBS for 20 min and incubated with 0.5% Triton X-100 in PBS for 15 min, followed by incubation with the mAb [at a concentration of 0.5 μg/mL diluted in 1% bovine serum albumin (BSA)] at 37°C for 1 h. After washing with PBS, cells were incubated with fluorescein isothiocyanate-labeled goat anti-mouse IgG (1:1000 dilution, 1% BSA) for 1 h in the dark. Cells were washed three times with PBS and nuclei stained with 4',6'-diamidino-2-phenyl-indole for 5 min. Fluorescence signals were detected using inverted fluorescence microscopy (Leica, Wetzlar, Germany).

### Primary screening for antigenic epitopes of HEV ORF3

Three pairs of primers were used to amplify truncated fragments of the ORF3 gene (Table 1) using the full-length ORF3 gene as the template. The products were inserted into pET-32a vector between the *Hind*III and *EcoR*I restriction sites. Plasmids were transformed into *E*.*coli* strain BL21 (DE3) plysS, and three recombinant proteins generated using standard expression and identification techniques. Interactions between the truncated ORF3 proteins and mAbs (at a concentration of 0.5 μg/mL) were detected via western blot, as described previously.

**Table 1:** Oligonucleotide primers used for amplification of ORF3 mutants. The lowercase letters in the universal sense primer represent the Kozak sequence and those in the antisense primers signify mutagenic nucleotides altering single P to A. N/A, not applicable.

**Primer**	**Sequence**	**Restriction site**	**Mutagenic nucleotides**	
universal sense primer		5ʹ-CCGGAATTCgccaccATGgcaCCACCATGCGCT-3ʹ	*Eco*R I	N/A
Anti-sense primers	Wild type	5ʹ-CCGCTCGAGACGGCGAAGCCCCAGC-3ʹ	*Xho*I	N/A
	P99A	5ʹ-CCGCTCGAGACGGCGAAGCCCCAGCTGGGGCAGATCGACGACGGGCGGGAGCGGagcGG-3ʹ	*Xho*I	cgg → agc
	P100A	5ʹ-CCGCTCGAGACGGCGAAGCCCCAGCTGGGGCAGATCGACGACGGGCGGGAGagcCG-3ʹ	*Xho*I	cgg → agc
	P102A	5ʹ-CCGCTCGAGACGGCGAAGCCCCAGCTGGGGCAGATCGACGACGGGagcGA-3ʹ	*Xho*I	cgg → agc
	P103A	5ʹ-CCGCTCGAGGGCGAAGCCCCAGCTGGGGCAGATCGACGACagcCG-3ʹ	*Xho*I	ggg → agc

### Phage display biopanning on HEV ORF3 monoclonal antibodies

Purified ORF3 mAbs were applied as the target and subjected to biopanning with the Ph.D.-12 Phage Display Peptide Library Kit (New England Biolabs, USA) according to instructions. For the first round, mAb at a concentration of 10 μg/well diluted in 0.1 M NaHCO_3_ buffer (pH 8.6) was coated onto 96-well plates (flat-bottom) overnight at 4°C. Next, the plate was blocked with PBS containing 1% BSA for 1 h at 4°C. Following six washes with TBST (50 mM Tris-HCl, pH 7.5, 150 mM NaCl, 0.1% Tween-20), the mAb was incubated with the original phage library diluted in TBST at a final concentration of 2×10^11^ pfu/mL (100 μL/well) for 30 min at room temperature with gentle shaking. Unbound phage was discarded via 10 washes with TBST. Bound phage was eluted using 100 μL elution buffer (0.2 M glycine-HCl, pH 2.2). Eluted products were neutralized with 15 μL of 1 M Tris-HCl (pH 9.1), and subsequently amplified in the host strain E.*coli* ER2738. Phages were purified using polyethylene glycol precipitation and titered.

Four rounds of biopanning were performed under similar conditions, except that the Tween-20 concentration in TBST was gradually increased to 0.5% (v/v). In each round, the ratio of the titer of amplified phage in input buffer (value of Input) to that in elution buffer (value of Output) was calculated to analyze enrichment efficiency.

### Sequence identification of genes encoding exogenous phage-displaying peptides

Individual phage clones were selected from the third and fourth rounds of biopanning, and amplified in *E*.*coli* ER2738, followed by precipitation according to the manufacturer's protocol. Genes encoding exogenous peptides of M13 were amplified with PCR using the sense primer 5ʹ-TCACCTCGAAAGCAAGCTGA and antisense primer 5ʹ-CCCTCATAGTTAGCGTAACG. PCR parameters were as follows: 95°C for 5 min, 30 cycles of 95°C for 30 s, 56.5°C for 30 s, 72°C for 30 s, and a final extension step at 72°C for 5 min. Deduced amino acid sequences were analyzed via post-sequencing and compared with that of swine genotype 4 HEV ORF3 protein (GenBank accession No. JX855794).

### Binding activity analysis of individual phages to monoclonal antibody

Binding activities of nine phage clones bearing different high-frequency peptides to ORF3 mAb were determined with ELISA. Briefly, ELISA plates were coated with ORF3 mAb at a concentration of 10 μg/well diluted in 0.1 M NaHCO_3_ overnight at 4°C. Plates were blocked with 1% BSA in TBS buffer (TBSB) at room temperature for 2 h. Following six washes with TBST, nine phages from the third and fourth rounds of biopanning at a concentration series from 10^10^ to 10^5^ pfu diluted in 0.1 M NaHCO_3_ were added to the plates for 1 h at 37°C, with the original mixed phage library as control. After six washes, M13 monoclonal antibody (Pharmacia, GE) at a dilution of 10^−3^ was added to wells for 1 h at 37°C. Subsequently, HRP-conjugated anti-mouse IgG antibody (dilution 1:5000, Sigma) was added and color developed using o-phenylenediamine (OPD). Optical density (OD_492_) was read using an ELISA plate reader. All experiments were performed in triplicate.

### Peptide synthesis

Candidate peptides from biopanning were synthesized by ChinaPeptides (Shanghai, China). BSA was conjugated to the N-terminus of each peptide. Purity of the peptide products obtained by lyophilization was >98%, as confirmed using high-performance liquid chromatography. Peptide sequences were confirmed via mass spectrometry.

### Identification of the specific epitope domain of ORF3 protein

The specific epitope domain corresponding to our ORF3 mAb was determined using ELISA. Briefly, five peptides (Table 2, part I) at a series of concentrations (100, 50, 10 and 0 μg/mL) diluted in 0.1 M NaHCO_3_ buffer were coated onto 96-well plates overnight at 4°C. BSA was used as the control. After blocking with TBSB, ORF3 mAb (at concentrations from 10^−3^ mg/mL to 10^−6^ mg/mL) was added to the plate, with normal mouse serum as the negative control. Subsequently, the HRP-conjugated anti-mouse IgG antibody was added and color developed using 3,3’,5,5’-Tetramethylbenzidine (TMB).

**Table 2:** Amino acid sequences of peptides synthesized in this study.

	**Sequence**	**length**
**Part I**	SAPPLPPVVDLPQLGL	16
	VVDLP	5
	SAPPLPPVVDL	11
	VVDLPQLGL	9
	SAPPLPPQLGL	11
**Part II**	SAPPLPPVVDLP	12
	SAA^99^PLPPVVDLP	12
	SAPA^100^LPPVVDLP	12
	SAPPLA^102^PVVDLP	12
	SAPPLPA^103^VVDLP	12

### Affinity analysis of the identified epitope with a single proline mutation in the proline-rich domain

ELISA and immunofluorescence analyses (IFA) were used to determine the influence of mutation of a single proline in the proline-rich domain on affinity of the identified epitope.

Four synthetic peptides with a single proline mutation (P →A) at different sites (Table 2, part II) were diluted in 0.1 M NaHCO_3_ (pH 8.6) and individually coated on a 96-well plate. The peptide with no proline mutations was used as the control. Affinities of these peptides for ORF3 mAb were detected with ELISA as above.

Meanwhile, four antisense primers were designed to obtain mutant plasmids. Recombinant pcDNA3.1-ORF3 was used as the PCR template to amplify mutant ORF3 genes using a universal sense primer and four different antisense primers (Table 1). Purified mutant gene fragments and pcDNA3.1 vector were digested with *EcoR*I and *Xho*I, and ligated to generate recombinant mutant ORF3-containing plasmids. Transfection of plasmids and IFA were performed as described previously.

### Establishment of peptide-based ELISA to detect positive serum

Two selected peptides were used as diagnostic reagents to detect anti-HEV-positive serum. Thirty-five positive and twelve negative sera, previously confirmed using commercial ELISA kit (Wantai Biological Pharmacy Co., Beijing, China), were maintained in our laboratory. Selected peptides (10 μg/well diluted in 0.1 M NaHCO_3_, pH 8.6) were coated onto ELISA plates overnight at 4°C, with the original mixed phage library as control. Wells were incubated with 5% BSA at 37°C for 2 h. After washing three times with PBST, positive or negative sera were added, followed by further incubation for 1 h at 37°C. Normal porcine serum was used as the negative control for this step. Plates were subsequently incubated with goat anti-pig IgG conjugated to HRP (KLP, USA) at 37°C for 1 h, and the same steps repeated.

## Results

### Construction and expression of recombinant plasmids

The full-length ORF3 gene of genotype 4 HEV originating from pig (accession No. JX855794) comprising of 345 bp (including initiation and termination codons) was predicted to encode a 114 amino acid protein. The HEV ORF3 sequences were successfully amplified and inserted into the appropriate expression vectors. Recombinant plasmids were analyzed via PCR, subjected to restriction enzyme digestion and verified with gene sequencing, which revealed no mutations (data not shown).

**Fig 1:**
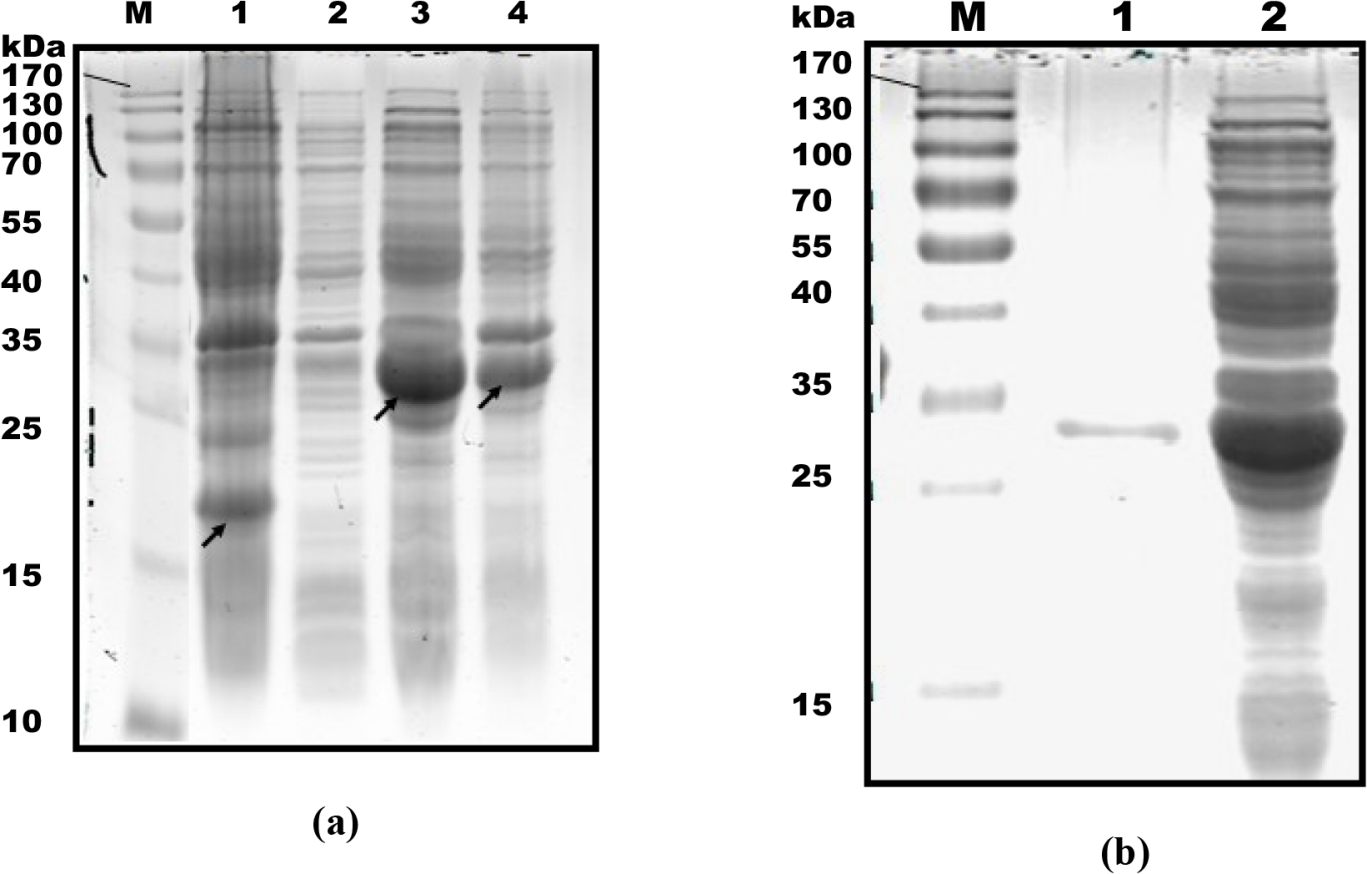
(a) Expression analysis of ORF3 protein at 5 h post-induction using SDS-PAGE. *Lane M*, protein molecular weight marker. *Lane 1*, pET32a empty plasmid. The arrow signifies His tag protein. *Lane 2*, uninduced bacteria transformed with pET32a-ORF3. *Lane 3*, deposit of lysed bacteria transformed with pET32a-ORF3. The arrow signifies ORF3 protein fused to the His tag. *Lane 4*, supernatant of lysed bacteria transformed with pET32a-ORF3. The arrow represents His-tagged ORF3 protein. **(b) Analysis of purified His-tagged ORF3 proteins.** *Lane 1*, purified ORF3 protein-tagged His. *Lane 2*, unpurified expression product.

Expression of the recombinant ORF3 proteins fused to the His tag was confirmed with 12% SDS-PAGE. Cells extracts from *E*. *coli* BL21 transformed with the recombinant plasmid after induction with IPTG presented an obvious band with a molecular weight of ~30 kDa corresponding to the expected size of the recombinant protein, compared to control bacterial cells. The results further indicate that the His-tagged ORF3 protein is in the soluble form (Fig 1A).

### Purification of recombinant protein

SDS-PAGE findings verified successful purification, since a clear band at ~30 kDa corresponding to the expected size of recombinant proteins was visualized (Fig 1B). Results were further confirmed with western blot using anti-HEV ORF3 polyclonal serum prepared and stored in our laboratory (data not shown).

### Preparation and identification of monoclonal antibodies to ORF3 protein

Bacterially expressed His-tagged HEV ORF3 protein was purified and used to immunize BALB/c mice. His-tagged ORF3 proteins were used to screen positive mAbs produced by mouse splenic cells fused with SP2/0 myeloma cells and the 6 x His tag protein used to negate the possibility of mAbs reacting with the histidine tag protein. ELISA analysis led to the identification of two monoclonal antibodies, mAb-1 and mAb-2, which reacted specifically with His-tagged ORF3 protein, and not the His tag (S1 and S2 Figs).

**Fig 2:**
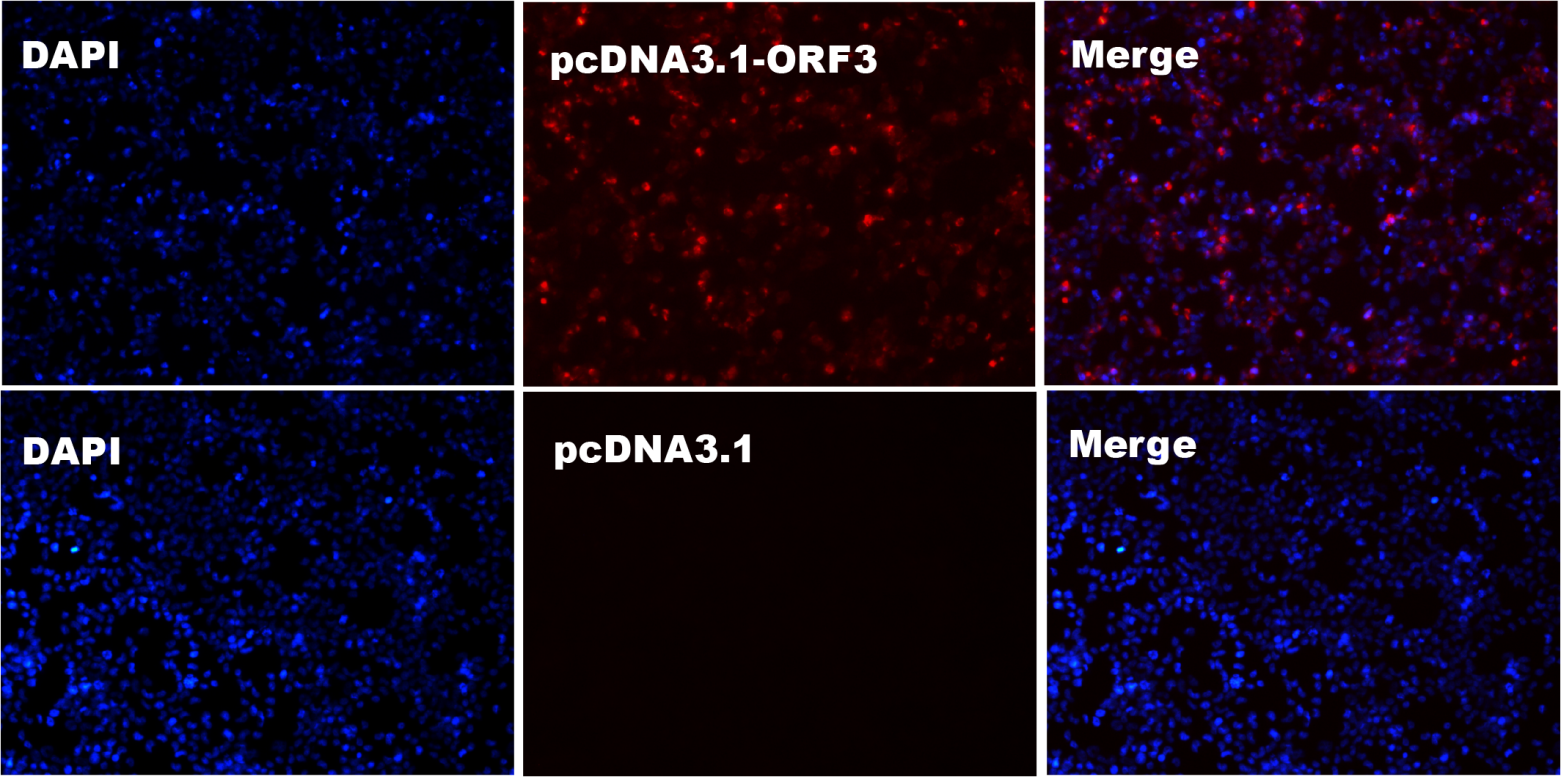
Detection of BHK cells transfected with pcDNA3.1-ORF3 plasmid via indirect immunofluorescence assay using mAb-1. BHK cells transfected with pcDNA3.1 empty plasmid were used as control. Nuclei were stained with DAPI. Results obtained with mAb-2 were similar to those with mAb-1 (S3 Fig).

The IFA confirmed the utility of the mAbs in identifying the HEV ORF3 protein expressed in BHK cells transfected with the pcDNA3.1-ORF3 recombinant plasmid. After transfection for 48 h, a bright red signal was observed in the cytoplasm, whereas no fluorescence was detected in mock cells transfected with empty pcDNA3.1 vector (Fig 2).

### Epitope screening of HEV ORF3 via western blot

**Fig 3:**
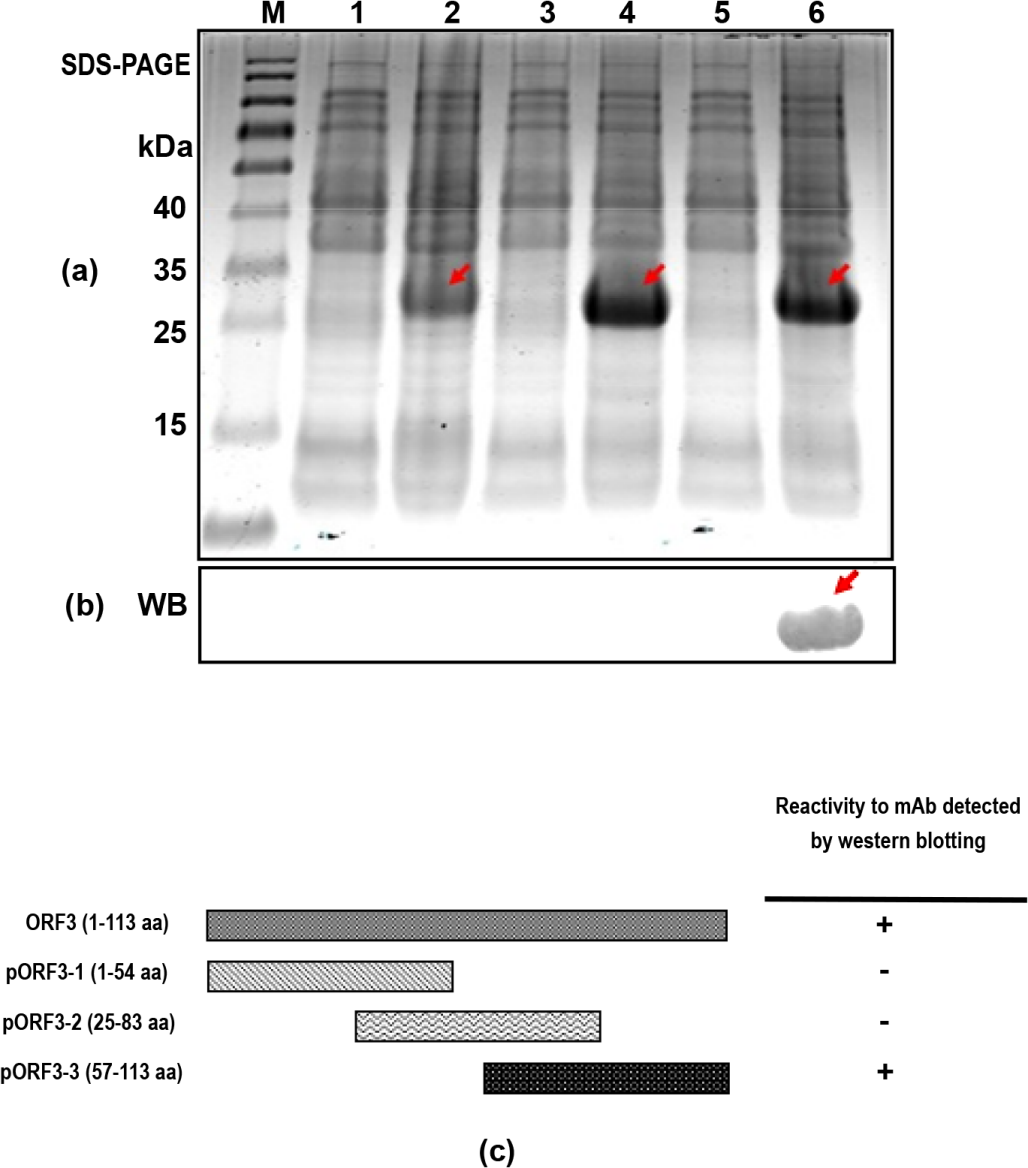
Expression of three truncated ORF3 proteins and identification of the antigenic epitopes. (a) Bacteria containing three truncated ORF3 genes induced by 1 mM IPTG at 37°C for 5 h and identified via SDS-PAGE. *Lane M*, protein molecular weight marker. *Lanes 1*, *3*, *5*, uninduced bacteria containing ORF3-1, ORF3-2 and ORF3-3 truncated genes, respectively. *Lanes 2*, *4*, *6*, induced bacteria containing ORF3-1, ORF3-2 and ORF3-3 truncated genes, respectively. Arrows indicate the positions of the three recombinant proteins with a molecular weight of ~27 kDa. (b) Identification of the antigenic epitopes of ORF3 using mAb-1 via western blot. Only the ORF3-3 protein was recognized by this mAb, as indicated with the arrow. The lanes in (b) are one to one correspond to lanes in (a), but experiments were performed using two SDS gels. (c) Schematic representation of the truncated HEV ORF3 constructs and reactivity to mAbs. The names and lengths of the truncated constructs are indicated. Binding ability to ORF3 mAb-1 was determined based on western blot results. “+”, positive result and “-”, negative result. Results obtained with mAb-2 were similar to those with mAb-1 (S4 Fig), and ORF3 protein recognized by two mAbs via western blot was shown in S5 Fig.

For preliminary screening of epitopes of the ORF3 protein recognized specifically by the prepared mAbs, the three truncated ORF3 proteins (ORF3-1, ORF3-2 and ORF3-3 encompassing the regions upstream, midstream and downstream of ORF3 protein, respectively) were successfully expressed (Fig 3A). Western blot analysis revealed that among the three proteins, only ORF3-3 containing the C-terminal region of the ORF3 protein was recognized by both mAb-1 and mAb-2 (Fig 3B and 3C).

### Biopanning to identify phages bearing peptides specific for ORF3 mAbs

To further confirm the range of the epitope domain, four rounds of phage display biopanning were performed using the prepared ORF3 mAbs as the target. The enrichment efficacies of eluted phages in each round were calculated. Our results showed that the enrichment efficacy values (Output/Input) increased gradually in the third and final rounds (Table 3), suggesting that the eluted phages obtained in these cycles display specific binding activity to ORF3 mAbs.

**Table 3:** Efficacy of biopanning for HEV ORF3 mAbs.

	**Titration value of round (pfu)**			
**Buffer of ratio**	**First**	**Second**	**Third**	**Fourth**
Input	10^11^	10^11^	10^11^	10^11^
Output	10^4^	10^5^	10^6^	10^7^
Output/Input	10^−7^	10^−6^	10^−5^	10^−4^

### DNA sequencing of selected phages

After four rounds of biopanning, a number of phages from the third and final rounds were selected randomly for sequencing using specific primers. Deduced peptide sequences encoded by genes from selected phages were analyzed. Nine peptide sequences comprising 12 amino acids each were identified in the last two rounds. Notably, similar peptide sequences were obtained after screening using the two mAbs, indicating that both antibodies recognize the same epitope. The peptide "SHHVVDLPIPYL" (SH) was shared by the majority of phages in the third and final round (Table 4). A consensus continuous amino acid motif "VDLP" was common to six groups of identified peptides (SH, SI, LE, HF, LN and ST), as determined using the MegAlign software program (DNASTAR, Inc., USA).

**Table 4:** Deduced amino acid sequences of peptides borne by selected phages. N/A, not applicable.

		**mAb-1**		**mAb-2**	
**Sequence**	**Sequence name**	**Third round**	**Fourth round**	**Third round**	**Fourth round**
SHH**VVDLP**IPYL	SH	20	11	22	21
NDALET**VD** **I** ^①^ **P**HH	ND	5	3	**N/A**	**N/A**
SIQGWPPA**VDLP**	SI	5	9	10	5
LEMT**VDLP**WSHN	LE	4	1	**N/A**	**N/A**
HFAWP**VDLP**LYQ	HF	3	1	4	2
LNMP**VDLP**HQKL	LN	3	6	6	2
EMD**II** ^②^ **DLP**YNLL	EM	**N/A**	**N/A**	5	3
STMLSHYA**VDLP**	ST	4	6	8	3
KAWQ**I** **D**TWDHLE	KA	2	1	**N/A**	**N/A**
**Invalid sequence amount**	**N/A**	**4**	**2**	**5**	**4**
**Total amount**	**N/A**	**50**	**40**	**50**	**40**

① Isoleucine (I) has similar structure and characteristics as Leucine (L)

② Isoleucine (I) has similar structure and characteristics as Valine (V). I, L and V all belong to the branched chain amino acid group. The underline indicates the continuous consensus sequence within the C-terminal region of ORF3 protein.

**Fig 4:**
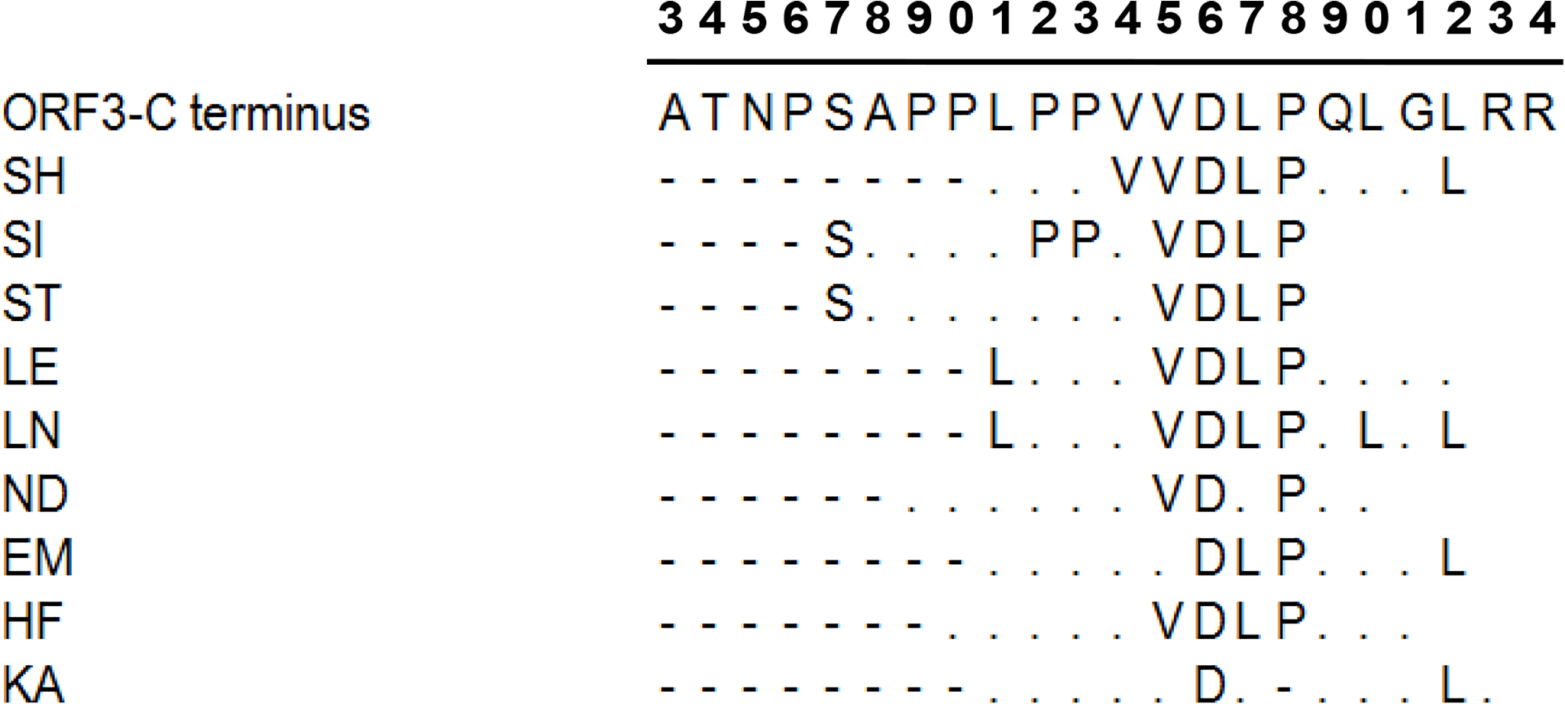
Comparative analysis between HEV ORF3 protein and peptides with deduced amino acids borne by selected phages. The consensus sequence was mainly identified in the ORF3 C-terminus. Only the sequence from A93 to R114 of ORF3 is presented. “.” represents residues of deduced amino acids differing from the ORF3 C-terminus, “-”indicates no residues. The numbers at the top signify the position of the residue at the ORF3 C-terminus. The first “3” represents position 93, and the last “4” represents position 114.

**Fig 5:**
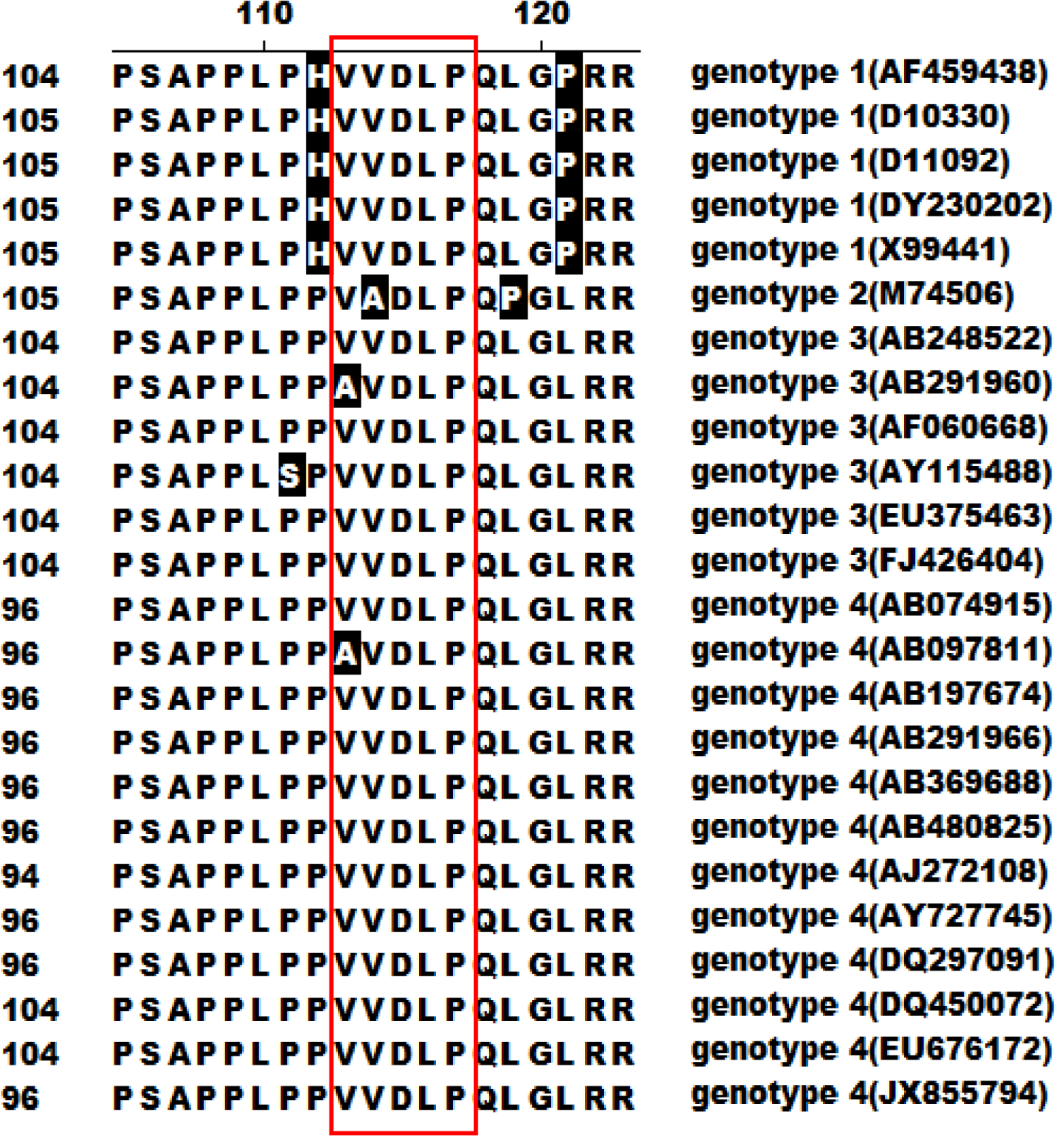
Comparative analysis of ORF3 C-terminal amino acid sequences among the four HEV genotypes. Residues shaded in black represent those differing from genotype 1 HEV ORF3. The consensus continuous sequence "VVDLP" is presented in the red box. The number in brackets represents the GenBank accession no. of the selected virus.

All nine peptide sequences were compared with ORF3 protein. The results showed that seven contained the consensus amino acids "DLP", coincident with ORF3 protein, and six contained the continuous sequence "VDLP". In addition, some peptides contained discontinuous consensus amino acids, such as S, P and L upstream and L downstream of "DLP", analogous to ORF3 protein. All consensus amino acids were present in the ORF3 continuous sequence (S^97^APPLPPVVDLPQLGL^112^) (Fig 4). Comparative analysis revealed that HEV ORF3 proteins of all four genotypes contain similar amino acid sequences in this fragment, with only slight differences, as shown in Fig 5.

### Confirmation of the binding ability of selected phages

**Fig 6:**
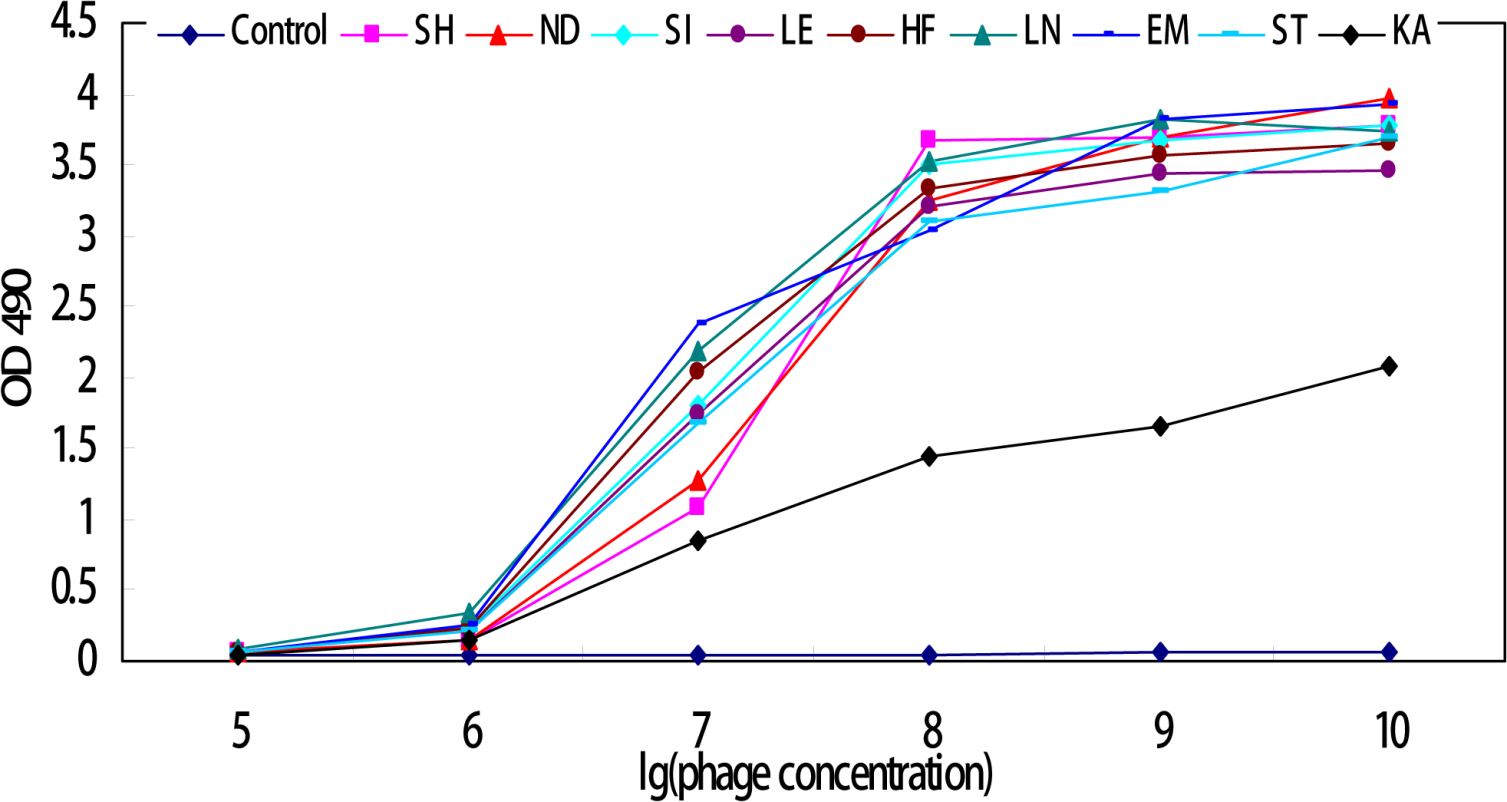
Binding analysis of selected phages to ORF3 mAb-1 using ELISA. Control and selected phage names are shown on the *z* axis, the OD_490_ values of individual phages and control on the *y* axis, and logarithm value of the phage concentration on the *x* axis.

Amplified products of the identified phages were analyzed for binding activity to mAb-1 using ELISA. Notably, phages bearing the consensus sequence "DLP" or "VDLP" displayed remarkable binding activity to mAb-1, compared to those with lower consensus sequences and the original mixed phage library. Binding activities of each positive phage were decreased in a dose-dependent manner, and minimum binding to ORF3 mAb achieved at a concentration of 10^5^ pfu per phage, which was not significantly different from the control (Fig 6).

### Influence of the nearby upstream proline-rich domain (PPLPP) on immunocompetence of the identified epitope

**Fig 7:**
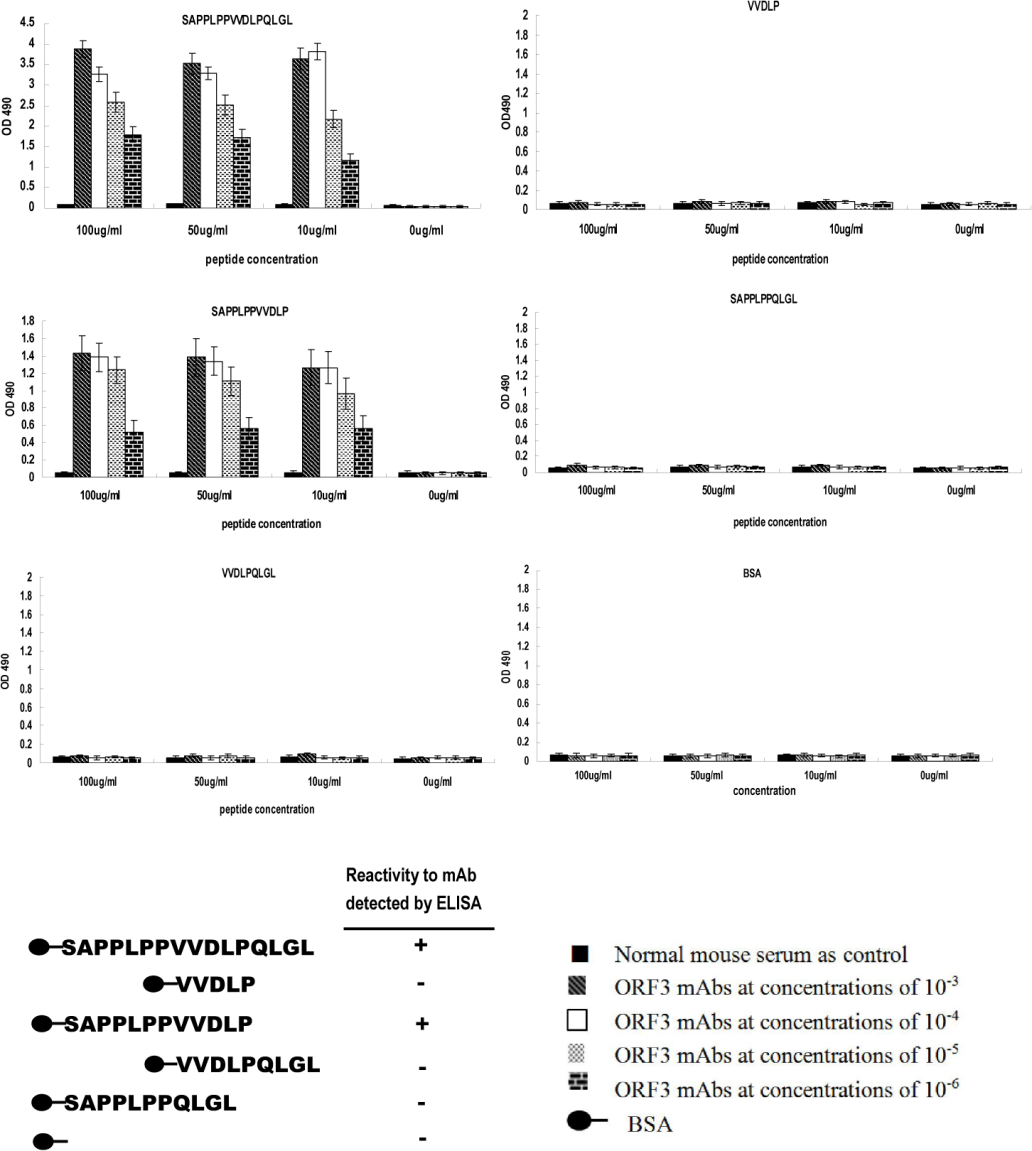
ELISA analysis of binding of synthetic peptides to ORF3 mAb. The binding activity of each peptide is presented in individual histograms. The *x* axis represents peptide concentration and *y* axis shows the OD_490_ value of each peptide at different concentrations. The color of each bar represents the ORF3 mAb serial concentration. Schematic illustration showing that only SAPPLPPVVDLPQLGL and SAPPLPPVVDLP react with the mAb,

Our results clearly indicate that the amino acid sequence S^97^APPLPPVVDLPQLGL^112^ of ORF3 represents an important antigen epitope domain within which V^103^DLP^108^ is the core site, since part or whole of this sequence presents a high frequency of occurrence in all positive phages examined. Accordingly, S^97^APPLPPVVDLPQLGL^112^ and V^104^VDLP^108^ were synthesized and binding activity with ORF3 mAb-1 determined using ELISA. Interestingly, the former displayed strong binding activity while the latter presented no activity, compared with control. Subsequently, three other peptides (SAPPLPPVVDLP, VVDLPQLGL, and SAPPLPPQLGL) were synthesized to analyze the influence of adjacent sequences on VVDLP binding to mAb. In ELISA experiments, SAPPLPPVVDLP possessed similar binding activity as S^97^APPLPPVVDLPQLGL^112^, while VVDLPQLGL and SAPPLPPQLGL presented no activity (Fig 7), suggesting that the upstream sequence of VDLP has a crucial effect on binding activity of the core sequence. In view of these findings, we hypothesized that the proline-rich domain influences the bioactivity of VDLP.

### Influence of proline mutations in the proline-rich domain on bioactivity of the identified VDLP epitope

**Fig 8:**
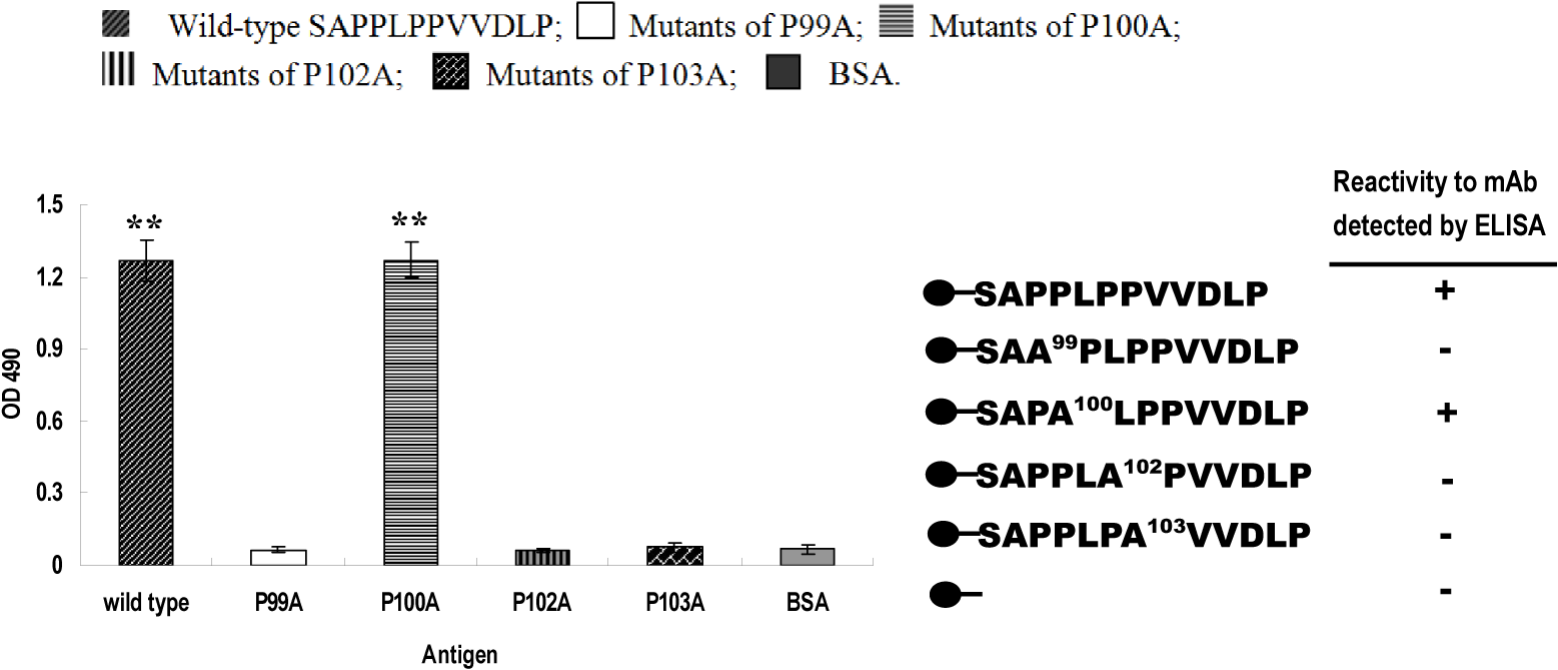
ELISA binding analysis of mutant S^97^APPLPPVVDLP^108^ peptides to ORF3 mAb. **A**ntigen names are shown on the *x* axis and OD_490_ value of each peptide on the *y* axis. Schematic illustration showing that the P100A mutant maintains similar binding activity to mAb as wild-type SAPPLPPVVDLP. “**” represents significant differences (*P*<0.01).

The S^97^APPLPPVVDLP^108^ peptide of ORF3 was used as the template, and individual P within PPLPP was replaced with A. Subsequently, four peptides (SAA^99^PLPPVVDLP, SAPA^100^LPPVVDLP, SAPPLA^102^PVVDLP and SAPPLPA^103^VVDLP) were synthesized to analyze the influence of single mutations in the proline-rich domain on VDLP. ELISA findings revealed that upon mutation of P^100^ to A (SAPA^100^LPPVVDLP), the peptide retained binding to ORF3 mAb-1, in contrast to P99A, P102A and P103A mutants (*P*<0.01), as shown in Fig 8.

Four mutant ORF3 gene-containing plasmids, whereby P at positions 99, 100, 102 and 103 was mutated to A, were constructed using pcDNA3.1 vector and transfected into Vero cells. IFA results showed that all four plasmids encoding the P to A mutations were detectable with ORF3 mAb-1 (S6 Fig).

### ORF3 peptide-based ELISA is less sensitive in detection of anti-HEV positive serum

The peptides SAPPLPPVVDLP and SAPPLPPVVDLPQLGL were selected for ELISA plate coating. Indirect ELISA was applied to detect anti-HEV positive serum. Only 14 out of the 35 positive serum samples confirmed with the commercial ELISA kit could be identified using the ORF3 peptide-mediated ELISA method, and both peptides had similar activity in reacting with anti-HEV positive serum. The negative serum samples confirmed with the commercial ELISA kit were consistently negative using peptide-based ELISA. The total coincidence rate between the two methods was 55.3% (Table 5), suggesting that ORF3 peptide-based ELISA developed in this study is less sensitive than the commercial diagnosis kit.

**Table 5:** Compliance test between commercial ELSIA kit and ORF3 peptide-based ELISA. +: positive no., −: negative no.

**Method**		**Commercial ELISA kit**		
		**+**	−	**Total no. detected by peptide-based ELISA**
**Peptide-based ELISA**	**+**	14	0	**14**
	−	21	12	**33**
	**Total no. detected by Commercial ELISA**	**35**	**12**	47
	**Total coincidence rate(%)**	55.3		

## Discussion

In the present study, His-tagged HEV ORF3 proteins were expressed in the *E*. *coli* system. Since recombinant His-tagged protein was expressed in the soluble form, the Ni Sepharose 6 Fast Flow affinity medium was selected to purify the protein. BALB/c mice were immunized with His-tagged ORF3 protein and standard hybridoma techniques performed to produce mAbs. After fusion of SP2/0 myeloma cells with spleen cells isolated from immunized Balb/c mice, purified His-tagged ORF3 was used to screen positive hybridoma cells, and His tag alone used to exclude false positive results. Consequently, two mAbs were identified, which specifically reacted with His-tagged ORF3 protein but were not recognized by the His tag.

BHK cells were transfected with the pcDNA3.1-ORF3 plasmid and IFA was employed to determine the binding ability of the identified mAbs with ORF3 protein obtained from the eukaryotic expression system. Notably, ORF3 protein expressed in the cytoplasm was recognized by both mAbs with similar detection efficiency. Distribution of the HEV ORF3 protein in host cells was in accordance with previous findings [30, 31], suggesting that our two purified specific mAbs may serve as valuable reagents in further research on the biological function of ORF3 using subgenomic expression strategies.

An earlier study suggested that the ORF3 protein could be used as a potential diagnostic target to detect antisera against HEV, since it contains important antigenic epitopes [28]. However, the epitope distribution of ORF3 protein remains ambiguous to date. Here, we expressed three truncated ORF3 proteins, ORF3-1 including the N-terminal region, ORF3-2 containing the middle region, and ORF3-3 protein encompassing the C-terminal region. There was an overlap of 29 aa between ORF3-1 and ORF3-2, and 26 aa between ORF3-2 and ORF3-3. Western blot data revealed that both mAbs only recognized the ORF3-3 truncated protein, suggesting that the specific antigenic epitopes exist within this fragment.

Phage display biopanning was further applied to screen the exact range of the epitope domain. The phage display peptide library consists of a pool of billions of random 12-residue peptides, and provides an efficient and powerful way to identify epitopes of target proteins through protein-protein interactions [32,33]. In our experiments, the two mAbs were coated on ELISA plates as targets respectively. After four rounds of biopanning, we observed increased enrichment efficacy, implying that the binding activities of specific phages to mAbs are gradually elevated in the final round. Meanwhile, a number of phages bearing different peptides were identified. Phages bearing the same peptide sequence were obtained from biopanning experiments, indicating that both mAbs recognize the same epitopes of HEV ORF3 protein. DNA sequencing revealed that nine groups of phages among the selected phage clones from the third and fourth rounds of panning share the consensus peptide. The SHHVVDLPIPYL (SH) peptide was shared by the most phages, and a continuous VDLP motif commonly observed in six groups of identified peptides. The other two groups contained VDIP and IDLP, which are similar to VDLP, since residues V, L and I belong to the branched chain amino acid group with similar structures and characteristics. The consensus VDLP or VVDLP sequence was additionally found in the C-terminus of swine HEV ORF3 protein. Other discontinuous consensus amino acids, such as S, P and L, upstream and L downstream of "DLP" were coincident with the HEV ORF3 protein sequence. All the above amino acids were present in the continuous sequence domain (S^97^APPLPPVVDLPQLGL^112^) of genotype 4 HEV ORF3.

Interactions between selected phages and ORF3 mAb were analyzed using ELISA. The data showed that all nine groups of identified phages recognized ORF3 mAb, with weaker binding capacity of the group KA. This result may be explained by the lower number of consensus amino acids, relative to native ORF3 protein.

Based on biopanning results, five peptides were successively synthesized to identify the epitope specific for mAb. SAPPLPPVVDLPQLGL and SAPPLPPVVDLP reacted with the mAb, but not the other peptides, indicating that VDLP is the core site of the epitope. This assumption may be explained by the findings that (1) VDLP was identified using the phage display biopanning method, (2) VVDLPQLGL and SAPPLPPQLGL did not react with mAb, confirming that SAPPLPP and QLGL are not recognized and the sequence upstream of VDLP must have a crucial effect on binding activity.

A proline-rich sequence exists immediately upstream of VDLP. The proline-rich domains in the C-termini of ORF3 of genotype 3 HEV and Avian Strain are reported to be necessary for virion release from infected cells [30,34,35]. To identify the key proline residues influencing immunocompetence of VDLP, mutants of S^97^APPLPPVVDLP^108^ with a single proline substitution (P →A) at positions 99, 100, 102 and 103 were synthesized and binding to mAb examined using an ELISA affinity assay. Only the SAPA^100^LPPVVDLP mutant retained binding activity with mAb, indicating that P99, P102 and P103 have a crucial effect on the immunocompetence of VDLP. Furthermore, IFA was performed using four mutant ORF3 gene plasmids with a P →A substitution at positions 99, 100, 102 and 103 to detect their binding capacity with mAb. Interestingly, all mutant proteins were recognized by the mAb. This result indicates that the epitope corresponding to mAb may be conformational, with PPLPPVVDLP comprising only part of the necessary region.

We additionally developed a peptide-based ELISA assay using SAPPLPPVVDLP and SAPPLPPVVDLPQLGL to detect anti-HEV positive serum. SAPPLPPVVDLP and SAPPLPPVVDLPQLGL displayed similar activity in detection of anti-HEV positive serum from pig. However, compared with the commercial diagnosis kit, total coincidence rate was only ~55.3%, implying that our ORF3 peptide-based ELISA method is less sensitive. The commercial ELISA kit is coated with HEV ORF2 protein. Accordingly, we speculate that the two ORF3 peptides react more weakly with anti-HEV serum than ORF2.

In summary, we have prepared two specific mAbs against genotype 4 HEV ORF3 protein, which may be used as efficient reagents for detecting ORF3 protein using subgenomic expression strategies. Our results suggest that the continuous amino acid sequence, VDLP, in the C-terminal region of ORF3 is an important epitope of HEV ORF3, but additionally requires upstream elements for immunoactivity. Additionally, three prolines, P99, P102 and P103, in the proline-rich domain have a significant effect on the immunocompetence of VDLP. However, peptides SAPPLPPVVDLP and SAPPLPPVVDLPQLGL containing the identified epitope display less sensitive detection of anti-HEV serum than the commercial ELISA diagnosis kit. Further studies are required to elucidate whether other epitopes exist in ORF3 protein and if the VDLP epitope is involved in additional important bioactivities, such as virus replication or virus-host interactions.

## Supporting Information

S1 Fig ELISA analysis to identify mAb-1. P/N value = (OD490_value_ORF3—OD490_value_Blank)/ (OD490_value_His tag—OD490_value_Blank), P/N value > 2 was designated as positive.
(TIF)

S2 Fig ELISA analysis to identify mAb-2. (TIF)

S3 Fig Detection of BHK cells transfected with pcDNA3.1-ORF3 plasmid by IFA using mAb-2. (TIF)

S4 Fig Identification of the antigenic epitopes of ORF3 using mAb-2 via western blot. *Lanes 1*, *2*, *3*, induced bacteria containing ORF3-1, ORF3-2 and ORF3-3 truncated genes, respectively.
(TIF)

S5 Fig ORF3 protein recognized by two mAbs via western blot. *Lane M*, protein molecular weight marker. *Lanes 1 and 2*, western blot using mAb-1 and mAb-2, respectively.
(TIF)

S6 Fig IFA results of four pcDNA3.1-ORF3 plasmids encoding the P to A mutations detected by ORF3 mAb-1. (TIF)
