# Supplementary figures and images for: A Proline-Rich Domain in the Genotype 4 Hepatitis E Virus ORF3 C-Terminus Is Crucial for Downstream V105DLP108 Immunoactivity
Source: PLoS One. 2015 Jul 15;10(7):e0133282. doi: 10.1371/journal.pone.0133282 (PMC4503470; doi:10.1371/journal.pone.0133282)

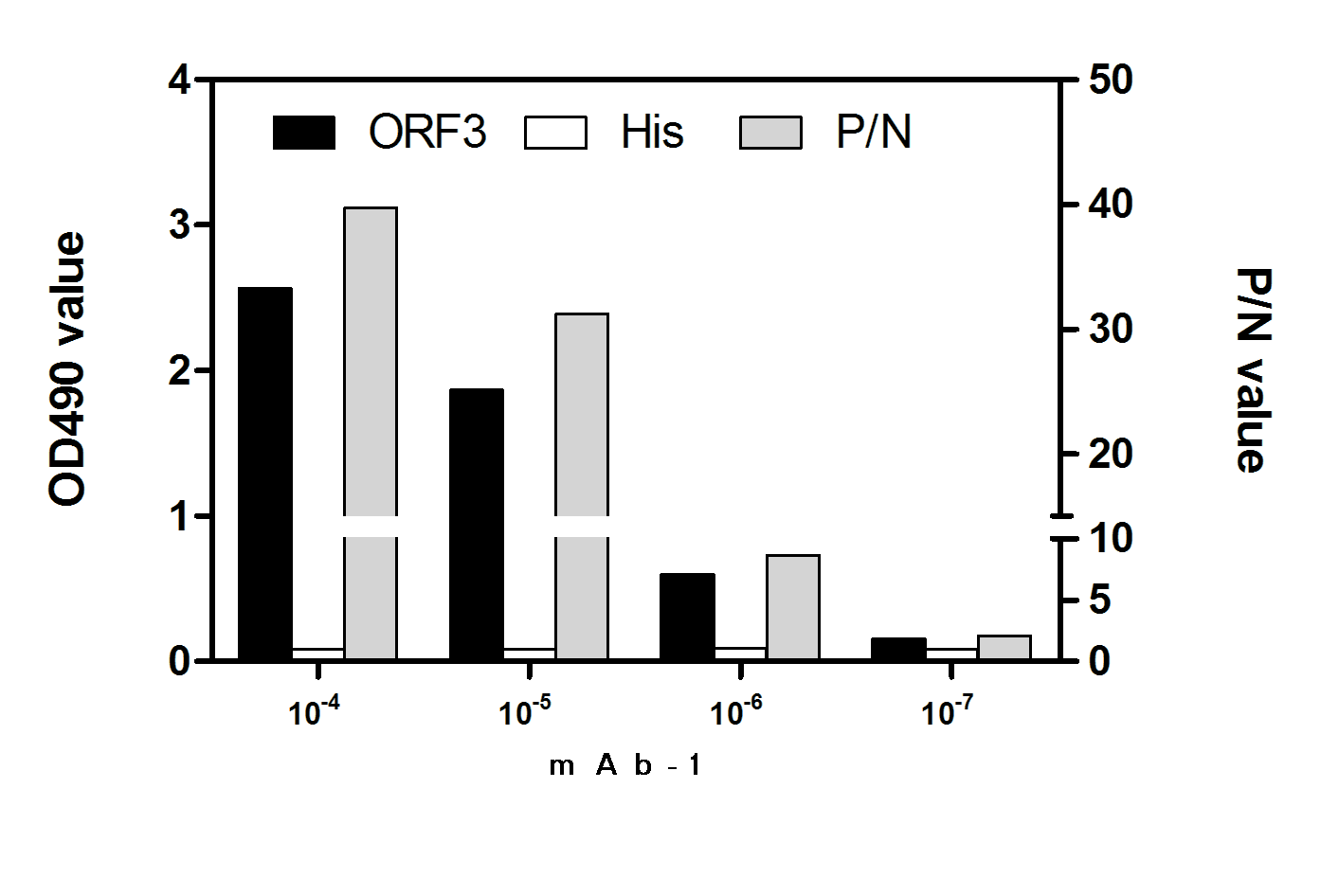

Supplement: S1 Fig — P/N value = (OD490valueORF3—OD490valueBlank)/ (OD490valueHis tag—OD490valueBlank), P/N value > 2 was designated as positive. (TIF) [file pone.0133282.s001.tif]

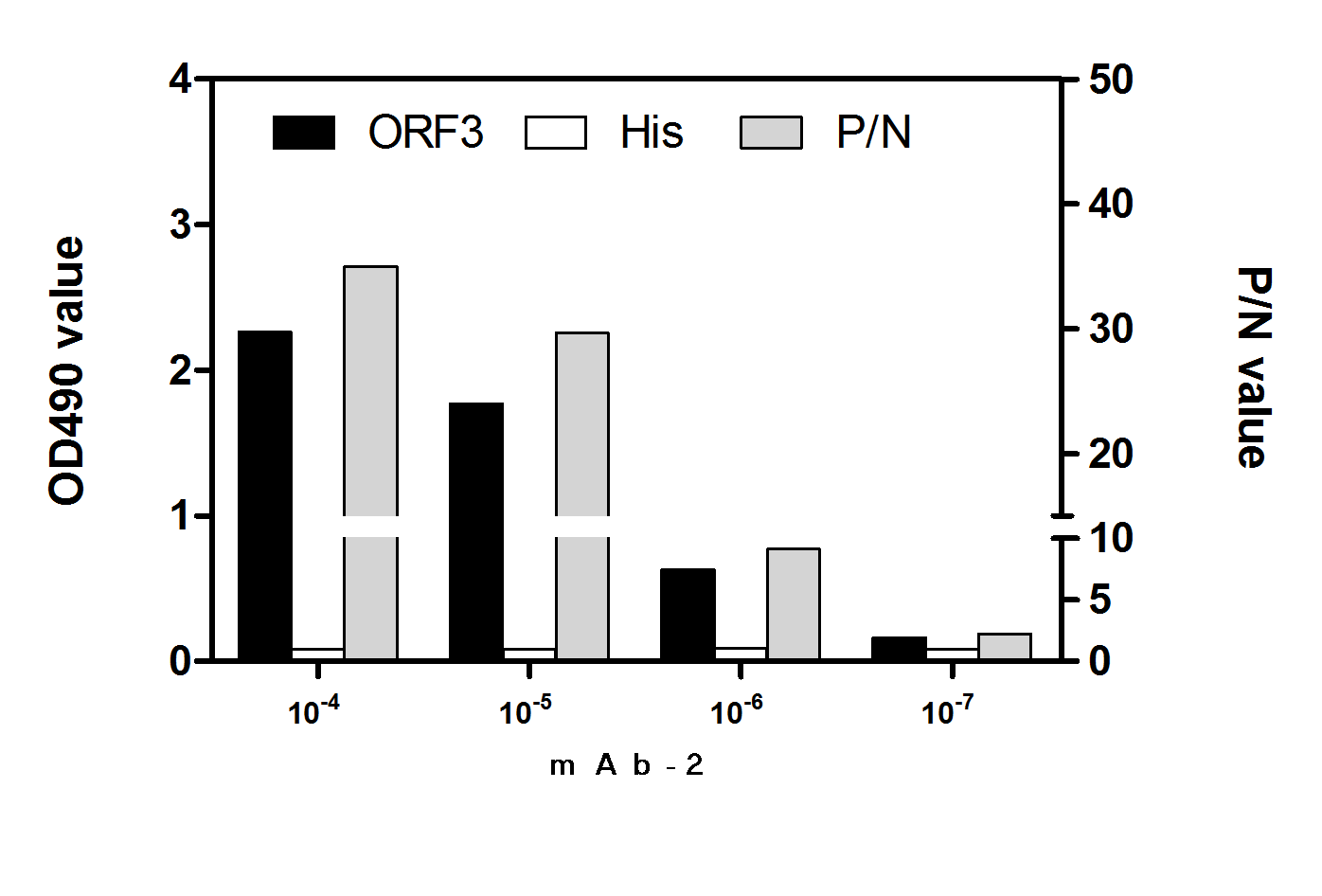

Supplement: S2 Fig — (TIF) [file pone.0133282.s002.tif]

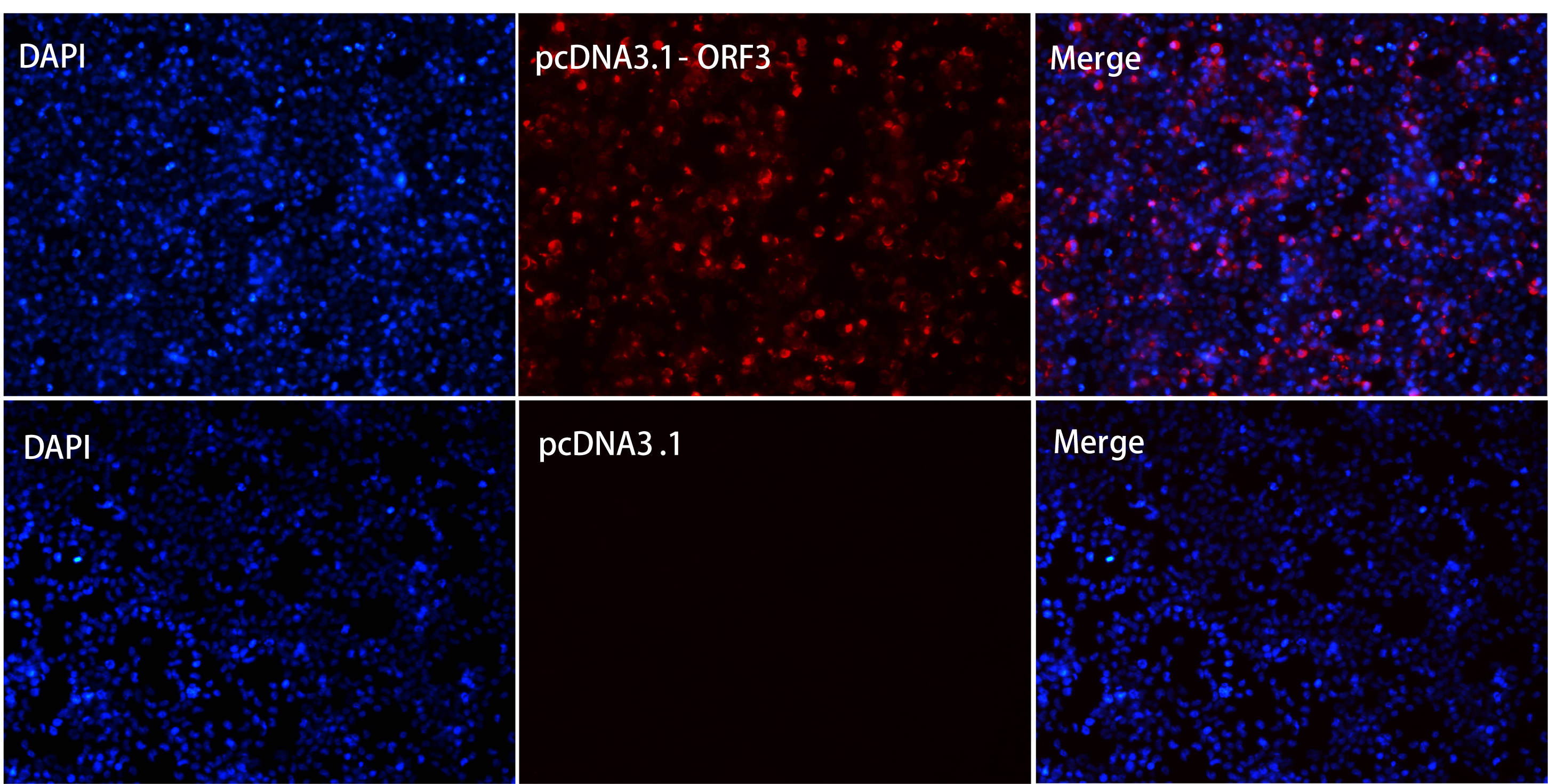

Supplement: S3 Fig — (TIF) [file pone.0133282.s003.tif]

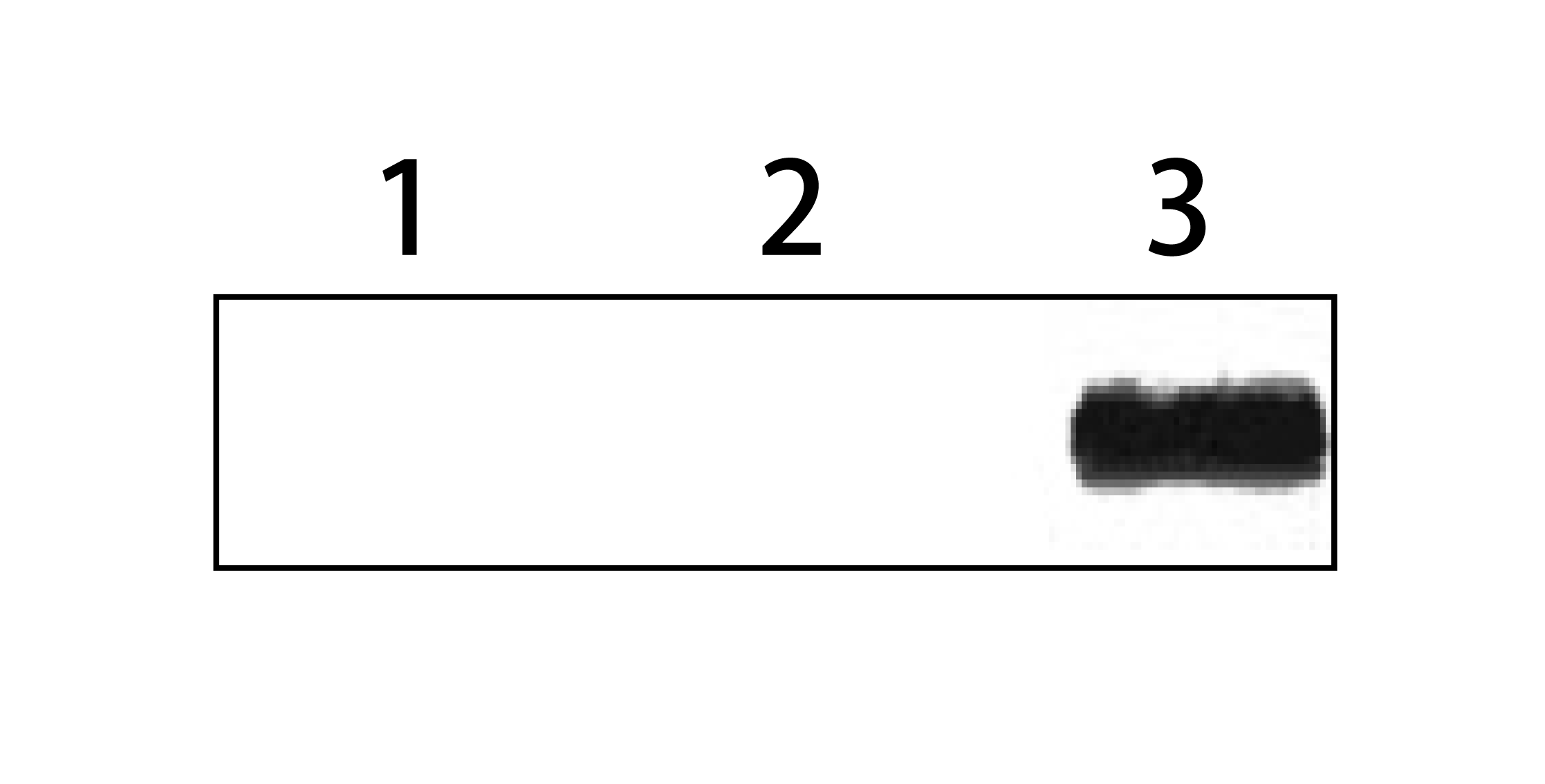

Supplement: S4 Fig — Lanes 1, 2, 3, induced bacteria containing ORF3-1, ORF3-2 and ORF3-3 truncated genes, respectively. (TIF) [file pone.0133282.s004.tif]

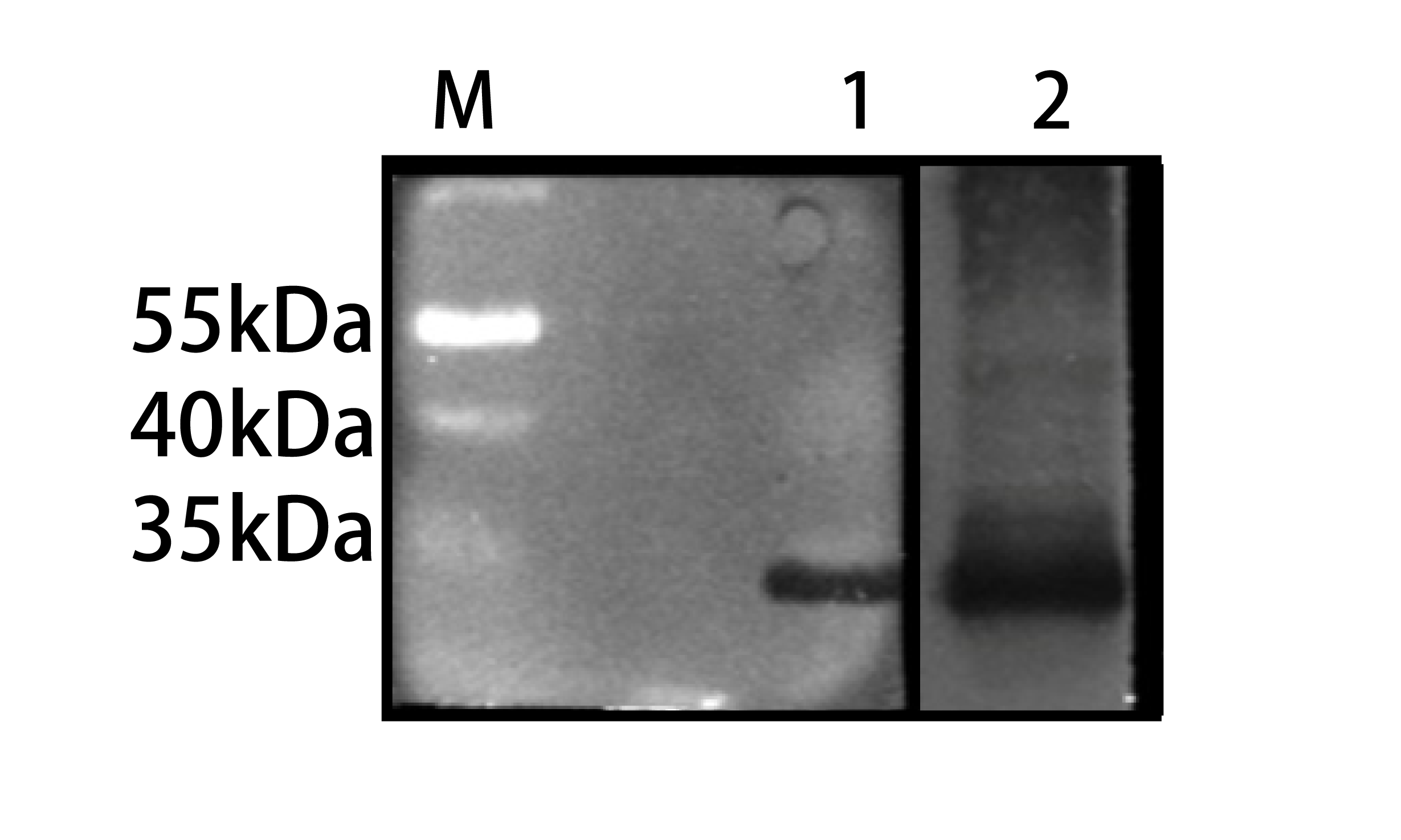

Supplement: S5 Fig — Lane M, protein molecular weight marker. Lanes 1 and 2, western blot using mAb-1 and mAb-2, respectively. (TIF) [file pone.0133282.s005.tif]

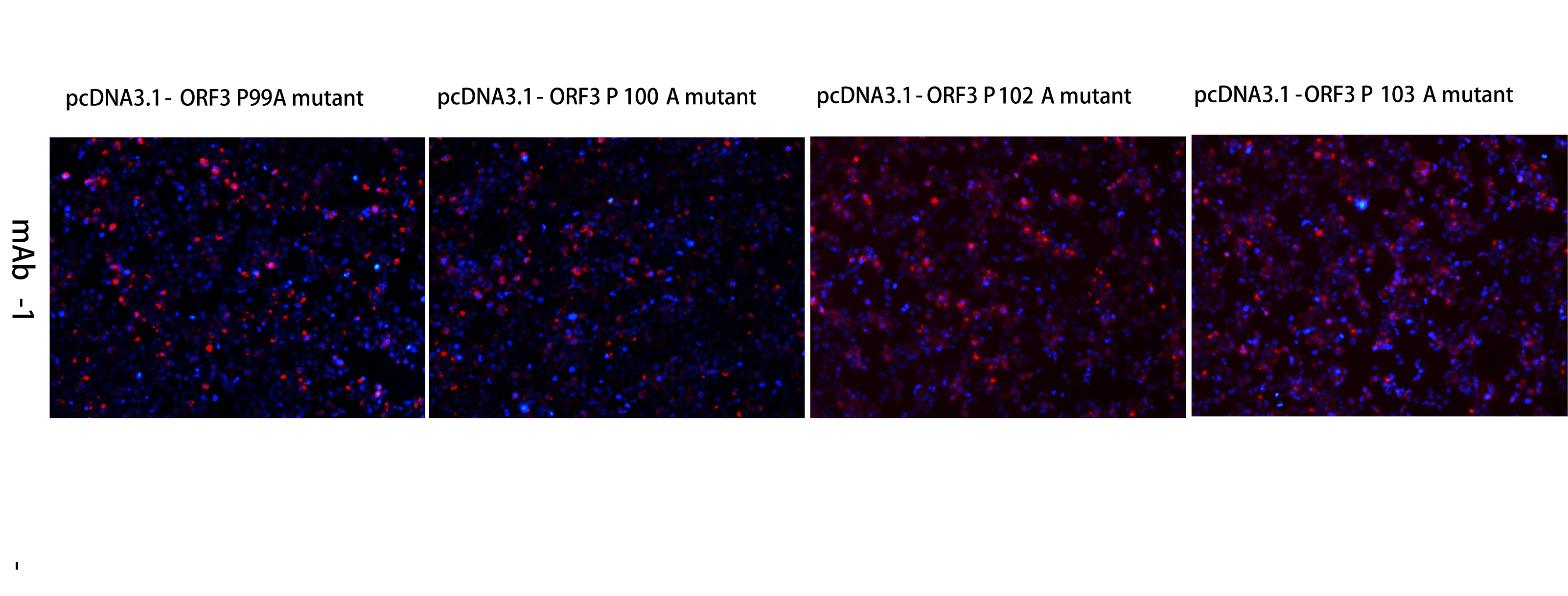

Supplement: S6 Fig — (TIF) [file pone.0133282.s006.tif]
